# Gut microbiota profiles are associated with different spontaneous cortical activity in healthy older people

**DOI:** 10.1038/s41598-025-16090-6

**Published:** 2025-08-27

**Authors:** J. Ignacio Serrano, Silvia Cruz-Gil, Cristina M. Fernández, Lara P. Fernández, Ana Sobrino-Santos, Isabel Espinosa-Salinas, Carolina Maestre, María-Jesús Latasa, Manuel Cebrián, Ricardo Ramos-Ruiz, Ana Ramírez de Molina, M. Dolores del Castillo

**Affiliations:** 1 Computational Models of Intelligence group, Center for Automation and Robotics (CAR, CSIC-UPM), Consejo Superior de Investigaciones Científicas (CSIC), Ctra. Campo Real km 0.200, 28500 Arganda del Rey, Spain; 2 Molecular Oncology and Nutritional Genomics of Cancer Group, IMDEA Food Institute, CEI UAM+CSIC, Carretera de Cantoblanco, 8, 28049 Madrid, Spain; 3 GENYAL Platform on Nutrition and Health, IMDEA Food Institute, CEI UAM+CSIC, Carretera de Cantoblanco, 8, 28049 Madrid, Spain; 4 Centro Nacional de Investigaciones Oncológicas (CNIO), C. de Melchor Fernández Almagro, 3, Fuencarral-El Pardo, 28029 Madrid, Spain; 5 Fundación para la Investigación e Innovación Biomédica del Hospital, Universitario Infanta Sofía y Hospital Universitario del Henares (FIIB HUIS HHEN), Paseo de Europa 34, 28702 San Sebastián de los Reyes, Madrid, Spain

**Keywords:** Gut-brain axis, Gut microbiota profiles, Spontaneous cortical activity, EEG source density, Healthy older population., Cognitive neuroscience, Microbiome, Cognitive ageing, Neurophysiology, Ageing, Microbiota

## Abstract

It has been shown that the gut and the brain are linked through a multimodal, bidirectional pathway called the gut-brain axis. In the gut-to-brain way, the gut microbiota has been shown to be the main regulator. In clinical practice, evidence of microbiota and brain interactions comes from the association of gut microbiota alterations with neurological and psychiatric conditions. However, until now, it remains unknown how the gut microbiota influences brain activity. In this paper, we show that different microbiota profiles from healthy older people are associated with different spontaneous activity in medial posterior cortical areas. These areas are associated with memory, language, and emotion processing abilities. Therefore, the results obtained provide evidence that non-pathological gut microbiota profiles are correlated to spontaneous cortical activity associated with cognitive functions that typically deteriorate with age. This implies that early nutritional interventions that modify microbiota composition could help delay or ameliorate natural age-related cognitive decline.

**Supplementary Information:**

The online version contains supplementary material available at 10.1038/s41598-025-16090-6.

## Introduction

During the last decades, a bidirectional relationship between the brain and the gut through what is called the gut-brain axis has been widely evidenced. In this regard, the gut microbiota has been shown to be altered in many Central Nervous System (CNS)-related co-morbidities. However, is there a relation between gut microbiota and brain activity in healthy conditions in any way?

The gut microbiota is the set of microorganisms that reside in the intestinal environment. The gut microbiota is considered an organ of the gastrointestinal tract that provides additional genes and functions to the genetic resources of the human species and is involved in multiple physiological processes (nutrition, development, immunity, etc.)^1^. Several essential functions carried out by the microbiota, such as the transformation of non-digestible food components into absorbable metabolites, the synthesis of essential vitamins, the elimination of toxic compounds, the strengthening of the intestinal barrier, or the regulation of the immune system, demonstrate its importance^2^.

Human gut microbiota matures and changes throughout our life cycles, from childhood to old age, and is a fundamental element in protecting our health. When the adult stage is reached, there is a stable community specific to each individual with dominant and subdominant bacterial species, although the abundance of different bacteria may fluctuate in response to external factors (diet, medications, travel, etc.)^3^. Thus, gut microbiota has particularities and characteristics specific to each individual, and may vary according to genetic endowment, diet, drugs, geography, and interaction with the environment^4^.

In order to identify patterns related to both human healthy and pathologic conditions, a series of reproducible profiles of gut microbiome variation in healthy adults, known as enterotypes, have been established, which are composed of a number of characteristic bacterial groupings. Three normality enterotypes have been proposed, based on the co-occurrence of different genus of microorganisms where one genus stands out from the rest^5,6^: enterotype 1, being *Bacteroides* the prevalent indicator; enterotype 2, led by *Prevotella*; and enterotype 3, with abundance of *Ruminococcus*. Any other combination of maladaptive and pathogenic microorganisms, together with a decreased biodiversity, can be considered intestinal dysbiosis.

In recent years, the number of preclinical and clinical studies that find relationships between pathological changes in the intestinal microbiota and neurological (Parkinson’s disease, Alzheimer’s disease) and mental (depression, stress, autism) diseases has grown significantly^7–11^, giving rise to the theory that a balanced microbiota is essential to maintain a healthy state. All these diseases share chronic inflammation, likely resulting from microbial dysbiosis, which points to the intestinal microbiota as a key factor in the onset of these disorders^12^.

The idea that the gut microbiota may have a significant impact on the CNS and neuroinflammation and, consequently, on neurological and behavioral disorders, is framed within the gut-brain axis research domain. This axis represents the bidirectional pathway of communication between the gut microbiota and the CNS.

There are several studies focusing on the direction from the gut to the brain that analyze whether the intake of different products, which modify gut composition, can affect spontaneous brain activity, cognitive function, anxiety, or stress. In^13^^,^^14^, subtle changes in the resting electroencephalographic (EEG) signal of brain regions associated with memory, attention, and inflammatory response, among others, were identified as a result of a psychobiotic intake by healthy subjects. Another study in children, who were in intensive care at birth and took antibiotics, showed that they presented greater power in a low-frequency band of their electroencephalogram in contrast to other children without antibiotic exposure^15^. In the same line of research, in^16^, a review of the modification of EEG rhythms, in the most clinically relevant frequency bands, based on the alterations present in the intestinal microbiota of people with metabolic diseases (obesity, diabetes), is carried out.

A functional magnetic resonance imaging (fMRI)-based study showed that the activity of brain regions involved in the control of emotion and sensation processing can be modulated in healthy women with the consumption of probiotic-fermented milk for 4 weeks^17^. Some longitudinal studies have also shown that administration of multi-strain probiotics reduces depression and improves attention in healthy volunteers by modifying brain functional connectivity, processed from resting-state fMRI and diffusion tensor imaging (DTI)^18^.

All these reviewed works have as a common goal to examine brain activity changes in relation to microbiota changes resulting from some nutritional or pharmacological intervention. These scientific studies of the gut-brain axis provide evidence on the influence of the microbiota on brain and behavior through the combined application of both neuroimaging (fMRI, EEG, DTI) and microbiota sequencing techniques.

This paper first presents an exploratory study that intends to characterize the relation between spontaneous brain activity in healthy older people, that is, with no metabolic, intestinal, and neurological disease, and their gut microbiota profiles. In a second step, the cognitive functions associated with this activity will be analyzed in order to identify possible cognitive disorders. Positive findings would facilitate and promote further research on preventing, delaying, or attenuating the onset of these disorders through simple nutritional interventions that modulate the gut microbiota.

## Results

### Microbiota-based clusters

Three clusters were identified by the X-Means clustering algorithm (A, B, and C) based on the microbiota relative population (%) at the genus level, composed of 26, 22, and 6 participants, respectively. Table 1 shows the average (standard deviation) values of the demographic and depression-related variables, together with the statistics of the differences between them. In this sense, the clusters did not present any significant difference in age, sex, body mass index, depression- and anxiety-related scores between them. The blood tests and anthropometrical data are shown in Table S1. Statistically significant differences between clusters are present in scattered parameters such as mean corpuscular volume (MCV) (*p* = 0.003) and Mean Corpuscular Hemoglobin (MCH) (*p* = 0.004). Also, adrenaline values are significantly higher in group C compared with group B (*p* = 0.004).

**Table 1:** Average (standard deviation)/Percentage (#) values of the sociodemographic variables of the participants in the three microbiota-based clusters and the statistics of the main effect.

	Cluster A N = 26	Cluster B N = 22	Cluster C N = 6	Statistic	*p* value
Age	62.35 (6.04)	62.00 (5.24)	60.50 (6.19)	F(2,51) = .252	.778
Sex (female)	53.85% (14)	68.18% (15)	66.67% (4)	χ^2^(2) = 1.118	.572
BMI	27.08 (5.59)	26.55 (3.28)	25.90 (4.79)	F(2,51) = .181	.835
MMSE	34.08 (1.79)	35.18 (5.02)	33.33 (1.97)	F(2,51) = .928	.402
DASS Depression	1.27 (1.85)	2.27 (3.40)	.50 (.55)	F(2,51) = 1.555	.221
DASS Anxiety	1.15 (1.62)	1.64 (1.81)	.50 (.84)	F(2,51) = 1.267	.290
DASS Stress	2.92 (2.51)	4.55 (3.78)	2.00 (1.41)	F(2,51) = 2.520	.090
DASS Total	5.35 (4.69)	8.45 (7.78)	3.00 (2.37)	F(2,51) = 2.633	.082
OSQ Subjective Satisfaction	4.46 (1.53)	4.32 (1.64)	4.67 (1.51)	F(2,51) = .128	.880
OSQ Insomnia	17.54 (5.41)	18.36 (6.46)	15.33 (6.19)	F(2,51) = .620	.542
OSQ Hypersomnia	4.31 (1.72)	4.09 (2.02)	3.83 (1.17)	F(2,51) = .201	.819
OSQ Total	26.31 (5.39)	26.77 (6.11)	23.83 (6.97)	F(2,51) = .598	.554

BMI, Body mass index; MMSE, Minimental State Examination; DASS, Depression Anxiety Stress Scales; OSQ, Oviedo Sleep Quality Questionnaire; F, ANOVA statistic; χ^2^, Chi-squared statistic.

To test the influence of the X-Means criterion of the optimum number of clusters on the resultant distribution, the algorithm was also executed with fixed numbers of clusters of 2 and 4. In the former case, clusters A and B were grouped as one while cluster C remained the same. In the latter case, cluster A was split into two subclusters, and clusters B and C remained the same. Therefore, the inner distribution was kept regardless of the number of clusters.

### Microbiota differences between microbiota-based clusters

After obtaining the microbiota-based clusters, we conducted an analysis to determine the pairwise differences in bacterial relative abundance between them.

**Fig. 1:**
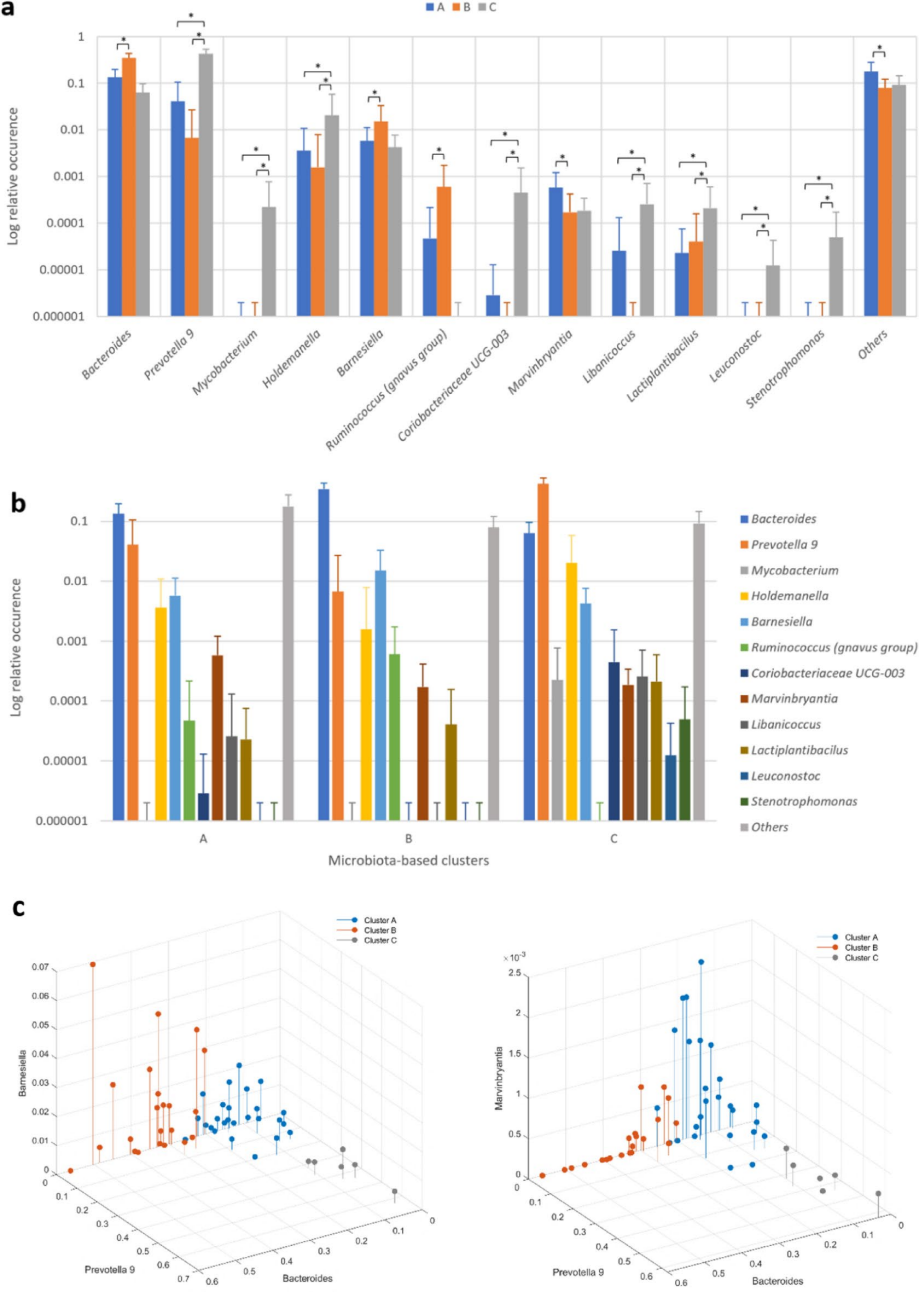
(**a**) Average log relative occurrence (%) of the bacterial genera that presented statistically significant differences between clusters A, B, and C after post-hoc correction. Brackets with an asterisk denote a statistically significant post-hoc difference between the linked clusters in the corresponding bacteria genus of the x-axis. Main effects: F(2,48) > 3.210, *p* < 0.49, N(A) = 26, N(B) = 22, N(C) = 6. (**b**) Microbiota diversity for the three clusters of the bacterial genera that presented statistically significant differences between them. Error bars denote standard deviation. (**c**) Scatterplots of the three clusters according to the relative abundance of *Bacteroides*, *Prevotella 9* and *Barnesiella* (left), and *Marvinbryantia* (right).

Figure 1a and Table S2 show the bacterial genera that presented significant statistical differences between clusters. According to Table S2, cluster B had a higher proportion of *Bacteroides* than clusters A and C. Besides, the proportion of *Prevotella 9*, *Mycobacterium*, *Holdemanella*, *Coriobacteriaceae UCG-003*, *Libanicoccus*, *Lactiplantibacilus*, *Luenostoc,* and *Stenotrophomonas* is higher in cluster C than in the other two clusters. Cluster B contained a higher proportion of *Barnesiella* and *Ruminococcus (gnavus group)* than cluster A, whereas cluster A presented a higher proportion of *Marvinbryantia* and other genera than cluster B.

Summarizing (Fig. 1b, c), the microbiota of cluster C is mainly composed of *Prevotella 9* and then *Bacteroides* in an inverse relation. The microbiota of cluster A is mainly composed of *Bacteroides* and then *Prevotella 9*. Despite the microbiota of cluster B being mainly composed of *Bacteroides* too (significantly higher than in the other two clusters), the second most abundant bacterial genus in this cluster is not *Prevotella 9*, like in cluster A, but *Barnesiella*. Cluster A, however, presents more abundance of *Marvinbryantia*. Moreover, the presence of *Mycobacterium*, *Leuconostoc,* and *Stenotrophomonas* was evident in cluster C, in contrast to clusters A and B. However, clusters A and B evidenced the presence of *Ruminococcus (gnavus group)*, in contrast to cluster C.

According to the three enterotypes defined by Arumugam et al.^5^ and Costea et al.^6^, the gut microbial composition of the clusters A and B found in the current study fits to the enterotype 1, since the genus *Bacteroides* is the most abundant, and cluster C meets the criteria defining enterotype 2, where *Prevotella* is the most abundant and is inversely correlated with *Bacteroides*.

### Spontaneous cortical activity differences between microbiota-based clusters

Analogously to the former section, we examined the pairwise differences in spontaneous brain activity between the previously obtained microbiota-based clusters. We performed this analysis for three EEG frequency bands (theta, alpha, and beta) and two conditions (eyes open and eyes closed) separately.

Tables S3 and S4 show the cortical areas of the Brainnetome atlas that presented a statistically significant main effect of cluster in eyes closed and eyes open conditions, respectively.

**Fig. 2:**
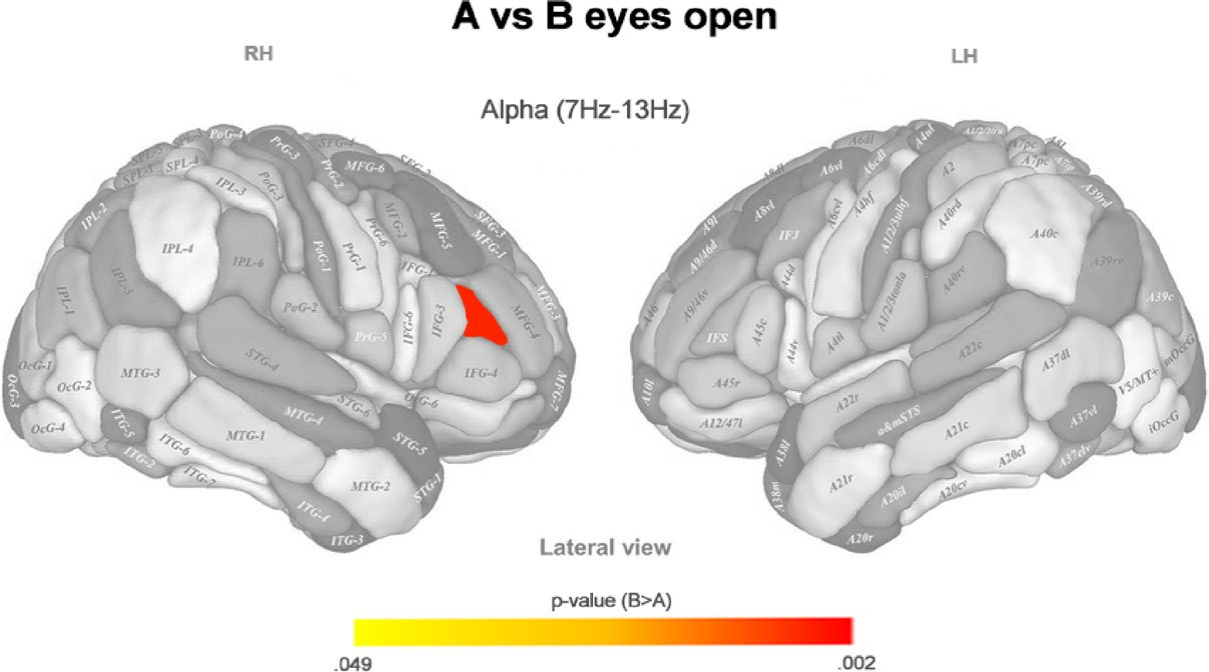
P color-coded values of the statistically significant differences in normalized EEG source density between microbiota-based clusters A and B after post hoc correction, in the eyes open condition for different brain areas (according to the Brainnetome atlas) and frequency bands. Main effect: χ^2^(2) = 9.131, *p* = 0.01, N(A) = 26, N(B) = 22. LH: Left hemisphere; RH: Right hemisphere.

The spontaneous brain activity between clusters A and B (Fig. 2) only differs significantly in the right inferior frontal sulcus, Brodmann areas (BAs) 9 and 46, in the low alpha band (B more active than A), in the eyes open conditions.

**Fig. 3:**
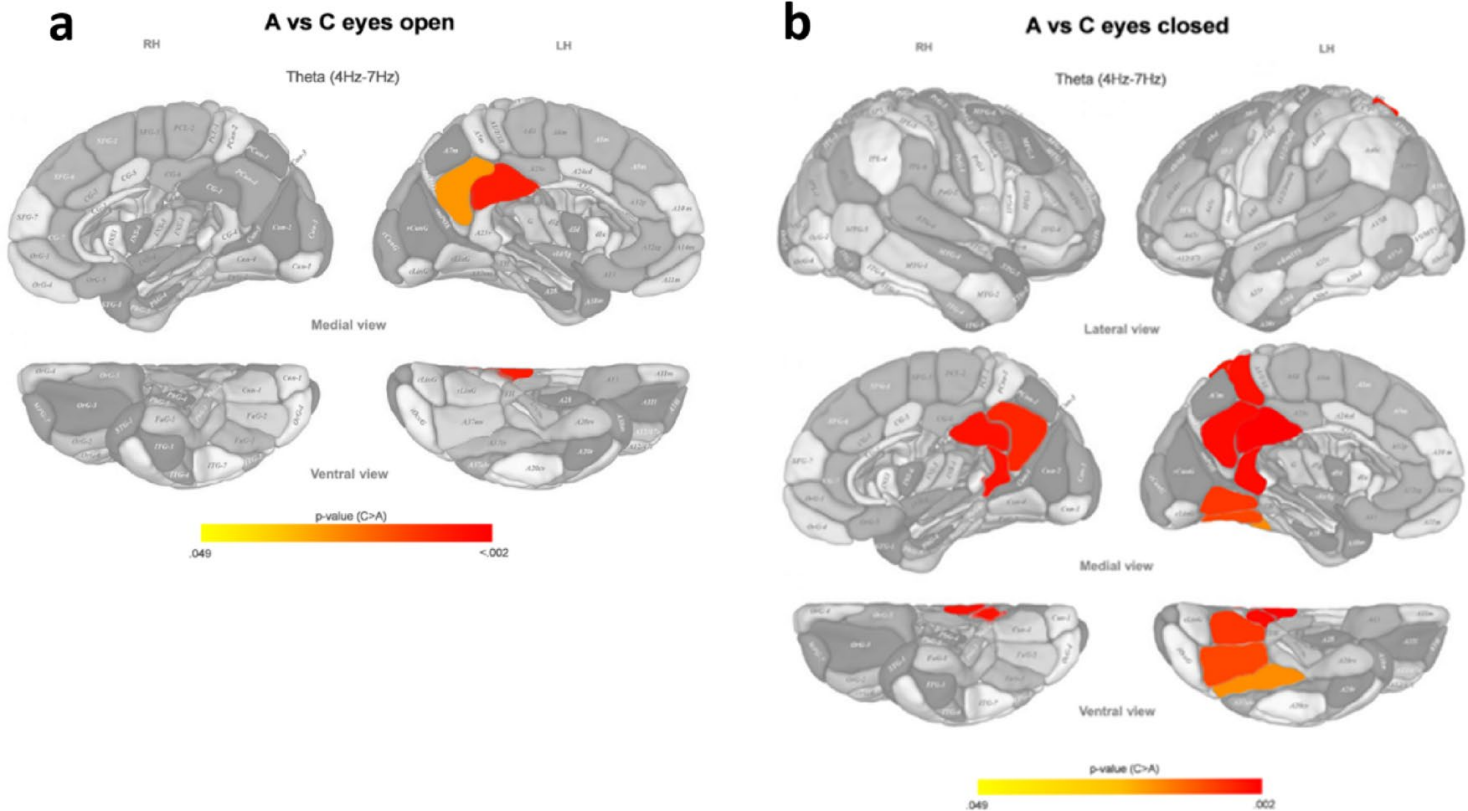
P color-coded values of the statistically significant differences in normalized EEG source density between microbiota-based clusters A and C after post hoc correction, in eyes open (**a**) and eyes closed (**b**) conditions for different brain areas (according to the Brainnetome atlas) and different frequency bands. Main effects: χ^2^(2) > 9.204, *p* < 0.01, N(A) = 26, N(C) = 6. LH: Left hemisphere; RH: Right hemisphere.

**Fig. 4:**
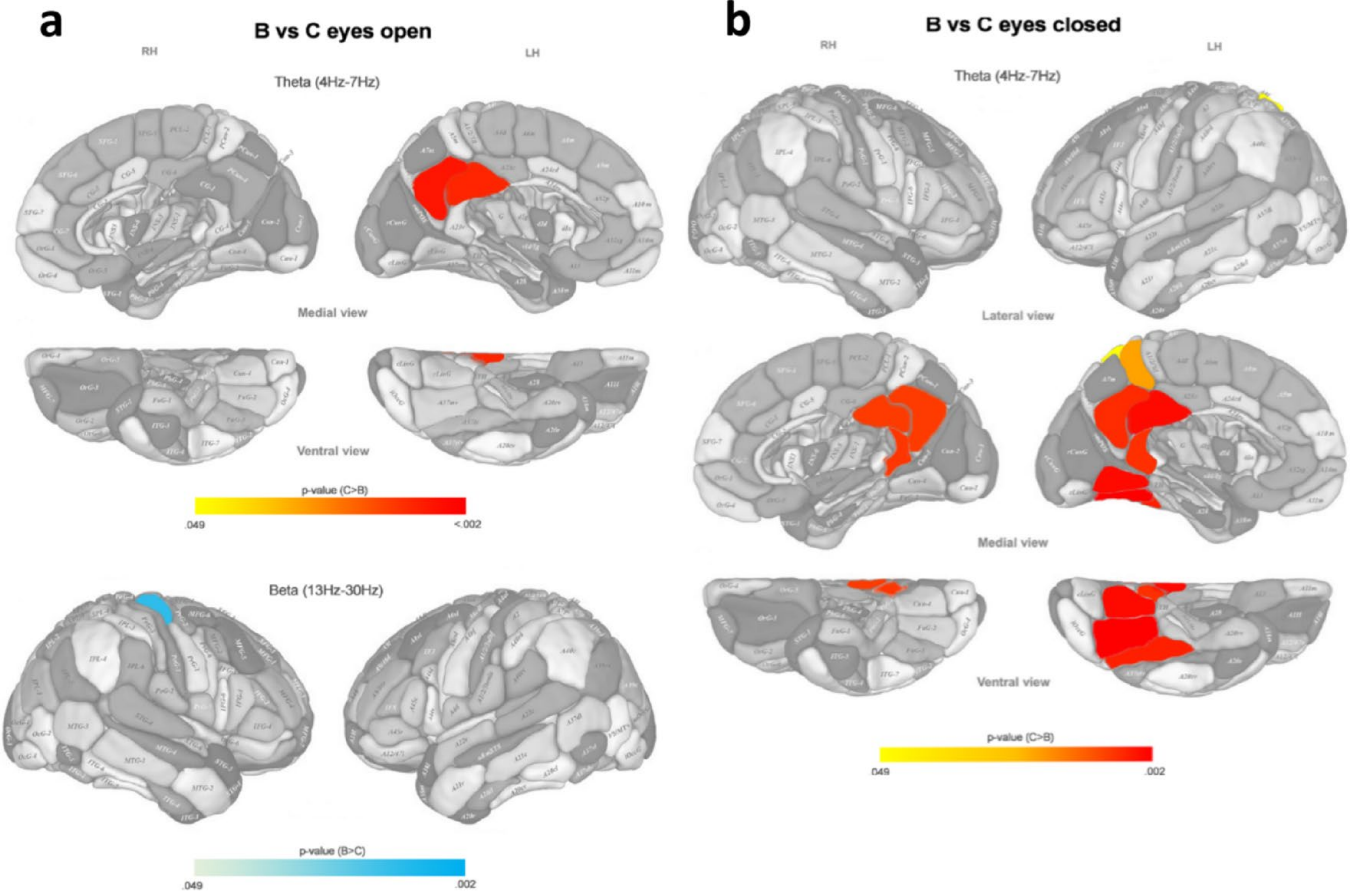
P color-coded values of the statistically significant differences in normalized EEG source density between microbiota-based clusters B and C after post hoc correction, in eyes open (**a**) and eyes closed (**b**) conditions for different brain areas (according to the Brainnetome atlas) and different frequency bands. Main effects: χ^2^(2) > 9.204, *p* < 0.01, N(B) = 22, N(C) = 6. LH: Left hemisphere; RH: Right hemisphere.

The differences of brain activity between cluster C, and clusters A and B were very similar (Figs. 3 and 4). Cluster C showed a higher activity than clusters A and B in the posterior cingulate cortex and the precuneus, bilaterally, and the left fusiform gyrus in the theta band during the eyes closed condition. During the eyes open condition, only the left posterior cingulate area and the left precuneus presented more activation in cluster C than in clusters A and B.

The particular difference between the brain activation of clusters C and B (Fig. 4) is the higher activity of cluster B in the right superior primary motor cortex (M1) in the beta band, during the eyes open condition.

### Cognitive functions associated with the areas with activity differences between clusters

Given that the EEG activity of the participants was recorded at rest, we cannot assign a functionality to the significant ROIs from our data. Consequently, we used a meta-analysis-based approach to identify the cognitive functions associated with those ROIs, the so-called activation likelihood estimation (ALE^19^), which computes a probabilistic estimation from the activation of brain foci in experiments reported in the literature.

**Fig. 5:**
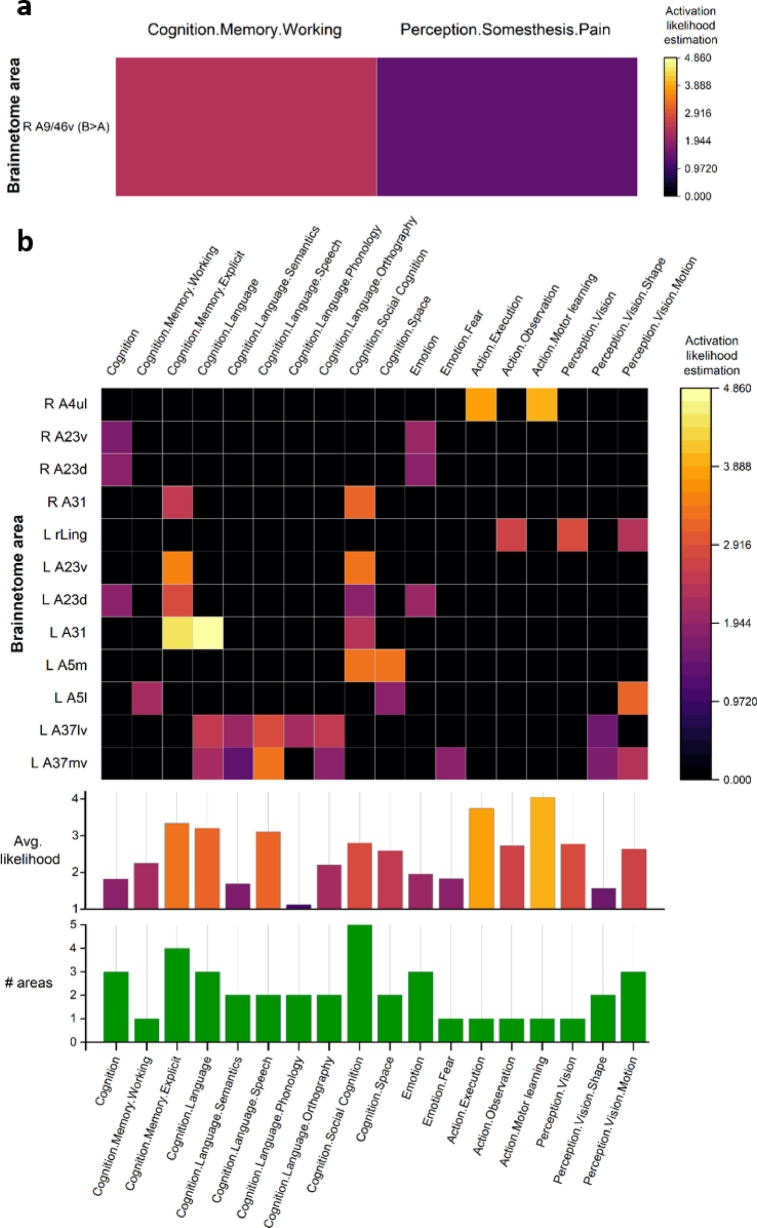
(**a**) Color-coded activation likelihood estimation (ALE) of the cortical areas (and average and number) that presented significant differences between clusters A and B; (**b**) cluster C and clusters A and B aggregated, during the tasks according to the brainmap.org taxonomy.

Figure 5 shows the ALE of the areas that significantly differed between clusters A and B (Fig. 5a), A and C, and B and C (Fig. 5b), during cognitive tasks according to brainmap.org. Note that Fig. 5b is a single figure for A-C and B-C differences because the ROIs affected in both cases are the same. Given the functional estimations, the only area significantly different in spontaneous activity between clusters A and B (Fig. 5a) is mainly activated during pain perception and working memory usage.

With respect to the areas with different activity between clusters A-B and C (Fig. 5b), they are also mostly related to episodic memory, language, and social cognition. However, other areas are also related to action perception and processing, cognition, and emotion processing.

This analysis reveals differences in memory, language, emotion processing, and action skills among the three microbiota profiles identified in the sample based on their associated spontaneous brain activity.

### Linear modelling of brain regions of interest (ROIs) activity from microbiota

In order to further test for the association between the gut microbiota abundance and the spontaneous brain activity in the ROIs presenting differences between clusters, we performed a linear modelling analysis of the sum of those ROIs’ activity, taking subsets of the relative abundance of microbiota genera as predictors.

**Fig. 6:**
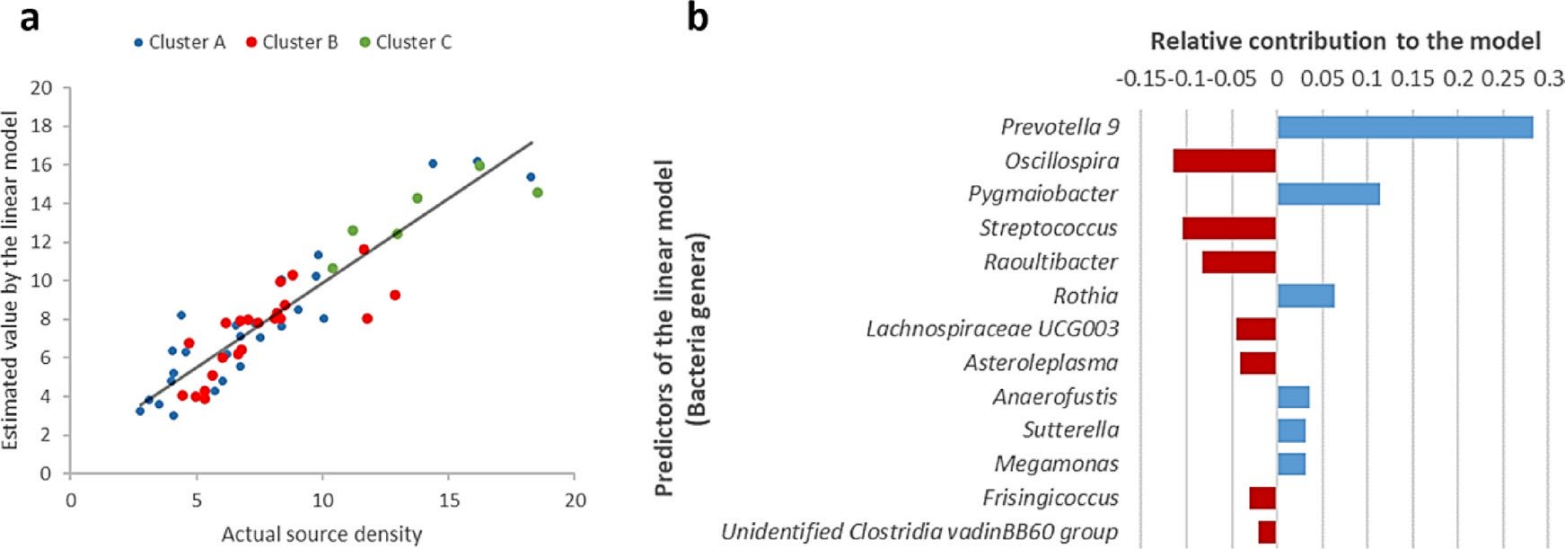
Linear model of the sum of the source activity of the ROIs that presented significant differences between clusters in the theta band with eyes closed. (**a**) Model fit. Colored points correspond to the different microbiota-based clusters. F(13) = 16.945, *p* < .0005, N = 54. (**b**) Relative contribution of each predictor to the model. Red negative bars indicate a negative coefficient, not a negative importance index.

Linear models of the sum of ROIs activity in the theta band eyes closed (Figs. 3b and 4b), theta band eyes open (Figs. 3a, and 4a), alpha band eyes open (Fig. 2), and beta band eyes open (Fig. 4a, bottom left) were constructed. The only significant model found was the one for the sum of the source density in the theta band, eyes closed, adjusted R^2^ = 0.846, F(13) = 16.945, *p* < 0.0005. Figure 6 shows the scatterplot (**a**) of the model fitting and the relative contribution of the predictors to the model (**b**). All the predictors contributed significantly to the model (*p* < 0.05).

The linear model indicates that the source density in the theta band during the eyes closed condition can be estimated from the abundance of some bacterial genera. The most contributing genus to the model was *Prevotella 9*, which in turn presented significant differences between clusters A and C, and B and C. This result points to the relative abundance of *Prevotella 9* as one of the sources of the difference in brain activity in the identified ROIs between clusters.

## Discussion

In this article, a study on healthy older subjects is presented in which the result of the analysis of their gut microbiota showed three groups with statistically significant differences in composition and abundance, mainly in the *Prevotella 9*, *Bacteroides*, *Mycobacterium*, *Holdemanella*, and *Barnesiella* genera. The analysis of their spontaneous electroencephalographical activity also showed significant differences between subjects in the three groups, especially between two of them (A, B) and the third one (C), covering the precuneus, the posterior cingulate cortex bilaterally, and the left fusiform area, all in the theta band. The hypothesis underlying the findings of this study on the observed differences in brain activity among the different groups of gut microbiota is that each gut microbiota profile could modulate differently the vagus nerve (VN) signal and, so, produce the specific brain activity found in each group. This hypothesis is supported by the scientific literature about the structure and dynamics of the VN, detailed in the next paragraphs.

Although the specific mechanisms of the interaction between the intestinal microbiota and the brain are still unraveled, the VN represents perhaps the most direct and fastest neural communication link between the gut microbiota and the brain^4^. Other major routes of this bidirectional axis are the immune system, which is activated by the microbiota and releases cytokines that circulate in the bloodstream and influence the CNS through the blood–brain barrier, and the neuroendoncrine system by regulating tryptophan metabolism and neurotransmitter production such as glutamate, butyrate, dopamine, serotonin and gamma aminobutyric acid (GABA), which are key neurotransmitters in many conditions^20^. Besides, microbial metabolites can also affect the brain dynamics directly, by producing, for instance, short-chain fatty acid (SFCA) that regulates neuroplasticity, or indirectly, by synthesizing some specific neurotransmitters and enzymes^21^.

There are preclinical and clinical evidences on the role of the VN in inflammatory processes that could contribute to the development of certain neurodegenerative diseases as Parkinson’s disease^22^ and mental disorders affecting emotion and cognition, such as depression^23^ or autism spectrum disorder^24^. On the other hand, vagus nerve stimulation (VNS) has demonstrated therapeutic benefits in CNS-related diseases such as epilepsy and depression^25^. The modulation of the VN, through stimulation and modification of the signal it transmits, might be the mechanism used by the gut microbiota to activate gut vagal afferent fibers and reach the brain. Several studies concerning the neurobiology involved in the response to VNS have revealed the existence of a neural circuit that can be considered as the afferent brain network of the VN signal^26^.

One of the key mechanisms to encode sensory information in the gut are by vagal afferent endings, located in the muscular layer and in the mucosa of the intestine, which are mechanical and chemical sensitive to gut stimuli or condition^27,28^. Accordingly, these terminals can detect and reply to gut muscular tone or inflammation^4^ and have an electrophysiological response to the presence of cytokines, nutrients, peptides, and hormones in the epithelial layer of the intestine. In addition, some bacteria can produce neurotransmitters such as serotonin, which, when absorbed, can act directly on the VN endings or, indirectly, through activation of enteric neurons^29^. Thus, either mechanically or chemically, the gut microbiota is involved in VN signaling^21^.

From the anatomical point of view, VN afferent fibers from the gut contact bilaterally with the Nucleus Tractus Solitarius (NTS)^25^. The physiological and functional role of the NTS is mainly based on the study of the vagus nerve stimulation (VNS) effects. A model of plausible vagal pathways implicated in the brain activity proposed in^30^, founded on several different noninvasive transcutaneous VNS (tVNS) fMRI studies on healthy people, confirms that, once VNS activates the structures of the lower brainstem as NTS, this one transmits activation to upper brainstem areas as the locus coerelus (LC) and dorsal raphe nucleus (DRN). The activation of LC produces norepinephrine that modulates large-scale brain networks involved in cognitive functions like memory, attention, emotion, or stress reaction. Also, a review of several studies contained in^25^ also validates how the vagal stimulation applied to either healthy or neurological impaired people impacts the LC activity, increasing the release of neurotransmitters as norepinephrine. At the cortical level, this pathway may reconfigure the activity of the insula, the dorsolateral and dorsomedial prefrontal cortices, and the sensory cortex. Furthermore, the activation of DRN produces serotonin, which selectively affects brain regions of the limbic system such as the ACC, the posterior cingulate cortex (PCC), and the dorsomedial prefrontal cortex (dmPFC). The PCC is one of the cortical areas involved in our study, whose activity differs between gut microbiota-based cluster C and clusters A and B.

Another anatomical model explaining the modulation of the brain activity and functional connectivity by tVNS is proposed in^31^. The model collects evidence from different studies with healthy participants showing that the stimulation of VN changes the activation in the insula, precentral gyrus, PCC, ACC, prefrontal and frontal cortices, S1, and parahippocampal gyrus. Again, this model shows that the PCC activation is affected by VN signaling.

Regarding the functional implications of the mentioned brain ROIs, VNS has also been studied to find its influence on cognitive functions in healthy people by analysing the neural pathways engaged in VNS action. In^32^, synchronized fMRI and tVNS were acquired to analyse the effects of VNS on brain activity and cognitive performance. This work showed that VNS results in a better performance on memory and language skills, and produces correlated significant spontaneous brain activity changes on the calcarine gyrus, fusiform gyrus, lingual gyrus, and parahippocampal gyrus. In addition, tVNS enhances recognition of emotions in faces^33^, resulting from the increased activity in the left fusiform gyrus^34,35^. In our study, this cortical region has a higher activity in cluster C.

In older healthy people, tVNS improves associative memory performance based on the hypothesis that tVNS modulates the memory using similar mechanisms to how emotion affects memory: emotional arousal increases the level of adrenalin that activates afferent VN fibers, and these activate the LC, which releases noradrenalin to the hippocampus and the amygdala that are brain areas involved in memory formation and consolidation^36^. Other neuroimaging studies revisited in^32^ point to the precuneus as a cortical brain area involved in visuospatial imagery, contextual memory, and information processing, among others, and to the parahippocampal gyrus as the main cortical input to the hippocampus. Both regions are significantly activated when tVNS is applied. In our study, the precuneus activity is also higher in the cluster C regarding clusters A and B.

Finally, the main areas that presented differences in spontaneous activity between microbiota-based clusters in our study, the precuneus and the PCC, are considered as a central hub part of the posterior default mode network (DMN)^37^. In the absence of pathology, these areas have been traditionally related to functions such as visuo-spatial imagery, episodic memory, and self-processing^38^, although the participation in working memory, reward, and fear processing has also been recently evidenced^39^. However, given their widespread functional and structural connections and their high relative metabolic rate^40^, any disorder in them might lead to a variety of cognitive changes^41^. As a matter of fact, the precuneus and PCC have been pointed out as key vulnerable areas for several neuropathologies and psychiatric disorders^39^.

Regarding the clusters A and B, the area that presented a difference in spontaneous activity between them, the right inferior frontal sulcus (IFS), BAs 9 and 46, is mainly implicated in working memory. Differences in cortical thickness in that area due to age were evidenced to correlate with working memory performance^42^. Given also that the IFS is an area differentially affected by ageing^43^, the different activity between clusters A and B could point to a difference in healthy brain ageing (despite no difference in chronological age). Again, this needs further research.

Caution is required when interpreting the results of our study. Cluster C is the one that presented more differences in microbiota and spontaneous EEG with respect to the other two clusters. However, cluster C is only composed of six participants. Despite the statistical significance is high for the differences found, a larger sample (especially for this cluster), and thus further research, would be needed to extrapolate the conclusion to the general coetaneous population. Nonetheless, cluster C is covering a singular set of participants in terms of microbiota abundance, since the selection of a different number of clusters (either lower or higher than 3) always yielded the same cluster C, and just split or grouped participants in clusters A and B. Although the findings were consistent, the sample size of this study was relatively limited, and we simply lacked power to detect additional associations. This may explain, at least in part, why associations of cluster C at the majority of biochemistry or anthropometric measures were not observed. Regarding MCH, MCV and adrenaline, differences in related parameters have been reported in the literature associated to some of the most predominant bacterial genera of the enterotypes described here^44,45^. Our results do not allow for any conclusions in this phase. A more comprehensive and detailed study of the role of each cluster in specific brain behaviors, catecholamines, and blood parameters would be needed. Besides, the assumption that different gut microbiota composition leads to signal-specific brain cortical areas in a distinct way mediated by the vagus nerve does not necessarily imply that gut microbiota is the sole cause of the brain activity differences, nor that the vagus nerve is the only link between gut microbiota and brain^20,46^. It is necessary to keep on making studies that include more factors that can have an impact on gut microbiota, brain activity, and behaviour, as pointed out in^47^. Moreover, since microbiota influences the brain through metabolites to a great extent^48^, an analysis of these could reveal and/or support the relation between the microbiota genera and the activation of the brain ROIs found in the present study. For instance, in our case, the elevated *Prevotella 9* in cluster C would shift SCFA production toward higher propionate levels, which can be 2–fourfold higher in *Prevotella* compared to Bacteroides-dominant clusters^49^. Propionate is a known precursor for hepatic glucose production, which has been reported to suppress feeding behaviour in ruminant studies through the stimulation of hepatic vagal^50^. This vagal signaling pathway could indirectly affect PCC and precuneus function through their connections with brainstem areas that receive vagal input, as commented above. Besides, the PCC presents a high rate of metabolism. In the human, cerebral blood flow and metabolic rate are ∼ 40% greater than average within the PCC and adjacent precuneus^40^. Given that propionate can serve as an energy substrate and acetate serves as an important energy source for host cells, particularly in the brain and peripheral tissues^51^, we could plausibly hypothesize that elevated propionate levels could potentially alter the metabolic activity in these highly metabolically active regions, thus explaining the differences in cortical activity between cluster C and clusters A and B. This, of course, would need further research to be confirmed.

Summarizing, this paper presents the first study to assess the relation between gut microbiota and spontaneous brain activity measured by resting-state EEG in healthy senior people. We have reported two main findings: (1) gut microbiota composition in healthy subjects could impact on brain activity, as suggested by two procedures based on a cluster analysis and on a linear model; and (2) the affected brain activity could influence the aging process of the memory, language, and social cognition, given the brain areas in which the brain activity showed differences.

## Methods

### Participant recruitment and sample collection

The recruitment was performed through the GENYAL Clinical Trials Platform at IMDEA Food Institute. This study was approved by the institutional Research Ethics Committee (protocol ID: IMD PI-055, IMDEA Food Foundation) and performed following the principles of research involving human subjects stated in the Declaration of Helsinki (1964) and Good Clinical Practice (GCP). All participants were clearly informed about the study methodology and provided written informed consent.

Anthropometric measurements, peripheral blood samples, and fecal samples were collected from a total of fifty-four healthy participants over 55 years of age (Male: 21, Female: 33). Sampling and data acquisition were performed between October and November 2022.

Inclusion criteria included: Age ≥ 55 years; BMI between 27 and 35 kg/m^2^; able to understand the informed consent; willing to comply with the study protocol. Exclusion criteria included: decreased cognitive function, pregnancy or breastfeeding, severe chronic health conditions (heart, liver, etc.), BMI > 35 kg/m^2^, or pharmacological treatment such as anxiolytics, antidepressants, medication for sleep disorders, or any type of psychotropic medication.

### Anthropometric measurements

Anthropometric measurements were collected following standardized methodology. Body Composition Monitor analyzer (BF511- OMRON HEALTHCARE UK, LT, Kyoto, Japan) was used to determine weight, % body fat, % skeletal muscle, and visceral fat, as well as basal metabolic rate. Height was measured with a stadiometer (Leicester-Biological Medical Technology SL, Barcelona). Waist and hip circumference (cm) were measured using Seca 201 (Quirumed, Valencia, Spain). Blood pressure and pulse were monitored with the Model M3 blood pressure monitor from OMRON HEALTHCARE UK, LT, Kyoto, Japan).

### Behavioral measures: sleep habits (OSQ questionnaire), emotional state (DASS-21 questionnaire), and mental state (MMSE questionnaire)

#### OSQ

Sleep habits were assessed via the Oviedo Sleep Questionnaire (OSQ)^52^. The OSQ is a validated interview that supports diagnosis of insomnia and hypersomnia during the previous month based on the DSM-IV and ED-10 criteria. The questionnaire is made up of a total of 15 items, including Subjective satisfaction with sleep, Insomnia (difficulty with falling or remaining asleep, early awakenings, or restorative sleep, among others), and hypersomnia (daytime sleepiness and its effects on daily tasks).

#### DASS-21

The psychological state of the participants was measured using the short version of the Depression, Anxiety and Stress Scale (DASS-21)^53^. Designed to assess the emotional states of depression, anxiety, and stress. The DASS-21 consists of 21 items, divided into three subscales of 7 Likert items. A score for each scale is calculated by summing all the related items, and these scores are later added to obtain a general score.

#### MMSE

The Mini-Mental State Examination (MMSE) (Spanish version) is a widely utilized cognitive screening tool designed to assess cognitive impairment and dementia^54,55^. The MMSE evaluates five cognitive domains: orientation, registration, attention and calculation, recall, and language. The total score has a maximum punctuation of 35, with lower scores indicating greater cognitive impairment.

### Fecal samples processing and 16S sequencing

#### Sample collection and DNA extraction

Fecal samples were sent to Novogene for 16S sequencing of the bacterial V3V4 region and further analyses: DNA was extracted by using the Magnetic Soil and Stool DNA Kit (TianGen, China, Catalog #: DP712), following the manufacturer’s protocol to ensure high-quality and high-yield DNA suitable for downstream applications.

#### PCR amplification of 16S rRNA gene

The 16S rRNA gene was amplified using a set of universal primers targeting the V3-V4 regions of the 16S rRNA gene. The primers used were (5′- CCTAYGGGRBGCASCAG-3′) and (5′- GGACTACNNGGGTATCTAAT-3′), which are known for their broad coverage of bacterial taxa while minimizing amplification of eukaryotic rRNA genes. The PCR conditions were optimized to ensure specific amplification, with an initial denaturation at 98 °C for 1 min, followed by 30 cycles of denaturation at 98 °C for 10 s, annealing at 50 °C for 30 s, and extension at 72 °C for 30 s, with a final extension at 72 °C for 5 min.

#### High-throughput sequencing

The amplified 16S rRNA gene fragments were performed by using specific primers connecting with barcodes. The PCR products of the proper size were selected through 2% agarose gel electrophoresis. The same amount of PCR products from each sample was pooled, end-repaired, A-tailed, and further ligated with Illumina adapters. Libraries were sequenced on a paired-end Illumina platform to generate 250 bp paired-end raw reads.

#### Data processing and analysis

Raw sequencing data were processed using the DADA2 pipeline, which includes quality filtering, dereplication, chimera removal, and sequence variant inference. This method allows for the recovery of full-length 16S rRNA gene sequences with single-nucleotide resolution and a near-zero error rate, ensuring high accuracy in microbial community profiling.

#### Taxonomic assignment and relative abundance calculations

Taxonomic classification of the ASVs (Amplicon Sequence Variants) was performed using the SILVA database, which provides comprehensive and up-to-date taxonomic information for 16S rRNA gene sequences. The classification was done at various taxonomic levels, including phylum, class, order, family, genus, and species.

By applying QIIME2’s classify-sklearn algorithm, a pre-trained Naive Bayes classifier is used for species annotation of each ASV. Then, the DADA2 method is mainly used for noise reduction.

According to the results of ASVs’ annotations and the feature tables of each sample, the species abundance tables at the level of kingdom, phylum, class, order, family, genus, and species are obtained. However, for posterior analysis species-level assignments were excluded for several reasons: (i) the sequenced 16S rRNA gene regions (V3-V4) lack sufficient variability to reliably distinguish closely related species, (ii) the available reference databases may not have comprehensive or well-curated species-level information for all taxa, (iii) species-level assignments can be noisy and prone to misclassification because of sequencing errors, natural intra-species variation, or horizontal gene transfer, (iv) Aggregating ASVs to genus level reduces sparsity (many zeros) in abundance tables, which improves statistical power and interpretability; and the genus-level assignment was chosen for relative abundance analyses.

The relative abundance of a given genus in a given sample is calculated as the number of detections of this genus in the sample over the total number of detections of all genera in that sample.

### EEG data acquisition

The participants were comfortably seated with their forearms resting on their thighs, one meter away from a white wall. They were asked to relax and breathe deeply with their mouth naturally open during three-minute periods, first with eyes closed and then with eyes open staring at the wall, with a one-minute resting interval between the two conditions. The EEG signal was acquired from 32 wet active ActiCAP electrodes (Brain Products GmbH, Gilching, Germany) placed on the scalp in the positions Fp1, Fp2, AF3, AF4, F7, F3, Fz, F4, F8, FC5, FC1, FC2, FC6, T7, C3, Cz, C4, T8, CP5, CP1, CP2, CP6, P7, P3, Pz, P4, P8, PO3, PO4, O1, Oz, O2 according to the 10–20 system. Reference was set to the Fz electrode and ground to the Fpz electrode. The signal was digitized and recorded by an actiChAmp amplifier (Brain Products GmbH, Gilching, Germany) at 256 Hz. The impedance of all electrodes was kept under 10 kΩ.

### EEG processing

The EEG signal was preprocessed offline by a custom script in MATLAB R2019b (The MathWorks Inc., Natick, MA, USA) using the EEGLAB^56^ functions. The signal was first cleaned by using Artifact Subspace Reconstruction (ASR)^57^, rejecting burst intervals above a threshold of 20 standard deviations (all the other parameters set to default). Then, the signal was bandpass filtered between 3 and 31 Hz. After that, eye- and muscle-related artifacts were removed by IClabel^58^ with thresholds above 70% of probability. Next, bad channels were interpolated using the default parameters of the PREP pipeline^59^. Finally, the signal was rereferenced to the average value among all channels.

Source density in six frequency bands (theta: 4 Hz–7 Hz; low alpha: 7 Hz–10 Hz; high alpha: 10 Hz–13 Hz; low beta: 13 Hz–18 Hz; mid beta: 18.5 Hz–21 Hz; high beta: 21 Hz–30 Hz) was calculated from the preprocessed signal by the eLORETA algorithm^60^ implemented in the LORETA-KEY software v20221229 (KEY Institute for Brain-Mind Research, Zurich, Switzerland). Source density was averaged for each of the 210 cortical areas defined by the Brainnetome atlas^61^.

### Data and statistical analysis

Participants were clustered from their microbiota population (at genus level) by the X-Means algorithm^62^, which automatically determines the optimum number of clusters (maximum intra-cluster, minimum inter-cluster similarity) based on the Bayesian Information Criterion (BIC). No standardization was applied to the data before clustering because we selected a non-scale-sensitive metric, the cosine distance, for measuring the similarity between clusters during the X-Means iterations, which guarantees that the clustering process is not affected by the difference of variance or range between the variables.

Differences in socio-demographic variables and microbiota population were tested by MANOVA tests, after confirmation of normality with the Shapiro–Wilk test and Q–Q plots. Post-hoc pairwise comparisons were performed with Tukey’s method after confirmation of a significant main effect of cluster. Differences in categorical variables were tested by the Chi-squared test. Significance was considered with *p* < 0.05. Effect size was reported as partial eta squared.

Differences in EEG source densities between clusters were tested by the Kruskal–Wallis test, after confirmation of non-normality by the Shapiro–Wilk test and Q-Q plots. Significance was considered with *p* < 0.01 in this case. Post-hoc pairwise comparisons were performed with Bonferroni’s correction after confirmation of the significant main effect of cluster. All the above analyses and tests were performed with SPSS v29.0.2.0 (Armonk, NY: IBM Corp.).

Finally, linear regression models were applied to predict the source density sum of the ROIs that presented significant differences between clusters. For that purpose, the automatic linear modelling procedure of the SPSS suite was applied, setting the number of predictors to the number of bacteria genera that presented significant differences between clusters, and the Akaike Information Criterion (AIC) as model fit index. Models with *p* < 0.05 (ANOVA) were considered statistically significant. The importance of each predictor was calculated as the residual sum of squares with the predictor removed from the model, normalized so that all the importance values sum up to 1.

## Supplementary Information

Below is the link to the electronic supplementary material.

Supplementary Material 1

## Data Availability

The datasets generated during and/or analysed during the current study are available from the corresponding author on reasonable request.
