# Supplementary material for: Gut microbiota profiles are associated with different spontaneous cortical activity in healthy older people
Source: Sci Rep. 2025 Aug 27;15:31590. doi: 10.1038/s41598-025-16090-6 (PMC12391352; doi:10.1038/s41598-025-16090-6)
Supplement: Supplementary file 1 — Supplementary Material 1 [file 41598_2025_16090_MOESM1_ESM.docx]

Table S1

|  | Microbiota-based clusters | | |  |
| --- | --- | --- | --- | --- |
|  | A (N=26) | B (N=19) | C (N=6) | P-value |
| Sex -Women  -Men | 12 (46.2%)  14 (53.8%) | 7 (36.8%)  12 (63.2%) | 2 (33.3%)  4 (66.7%) | 0.74 |
| Age | 62.4 (6.07) | 62.7 (4.93) | 61.5 (5.36) | 0.893 |
| Leukocytes | 6.16 (1.52) | 5.86 (1.32) | 5.44 (1.23) | 0.499 |
| Red blood cells | 4.76 (0.397) | 4.80 (0.443) | 4.68 (0.595) | 0.843 |
| Hemoglobin (g(dL) | 14.8 (1.28) | 14.2 (1.30) | 14.0 (1.93) | 0.25 |
| Hematocrit (%) | 43.7 (3.26) | 42.3 (3.76) | 42.0 (5.38) | 0.311 |
| MCV (fl) | 92.0 (3.65) | 88.1 (4.12) | 89.7 (1.06) | 0.003 |
| MCH (pg) | 31.1 (1.35) | 29.6 (1.67) | 29.9 (1.09) | 0.004 |
| MCHC (g/dL) | 33.8 (1.07) | 33.6 (0.631) | 33.3 (0.946) | 0.429 |
| RDW (%) | 13.1 (0.708) | 13.1 (0.678) | 13.0 (0.715) | 0.974 |
| Platelet count (103/uL) | 235 (53.3) | 238 (48.5) | 222 (34.2) | 0.714 |
| Platelet volume (fL) | 8.70 (0.862) | 8.63 (1.02) | 8.85 (1.08) | 0.703 |
| Lymphocytes (%) | 35.9 (9.34) | 34.4 (6.36) | 37.7 (4.75) | 0.289 |
| Monocytes (%) | 6.40 (1.45) | 6.32 (0.849) | 6.65 (0.683) | 0.682 |
| Segmented (%) | 54.0 (9.45) | 55.7 (6.16) | 51.8 (5.38) | 0.201 |
| Eosinophils (%) | 3.01 (1.75) | 3.04 (1.31) | 3.37 (1.75) | 0.707 |
| Basophils (%) | 0.681 (0.251) | 0.626 (0.231) | 0.500 (0.210) | 0.238 |
| HBA1C (%) | 5.81 (0.315) | 5.84 (0.302) | 5.65 (0.217) | 0.342 |
| HBA1C IF CC (mmo/mol) | 40.0 (3.45) | 40.4 (3.31) | 38.3 (2.37) | 0.342 |
| Homocysteine (umol/L) | 16.8 (2.47) | 18.3 (3.26) | 16.7 (3.85) | 0.196 |
| Glucose (mg/dL) | 90.5 (11.2) | 87.6 (8.49) | 87.3 (6.77) | 0.685 |
| Urea (mg/dL) | 38.4 (10.8) | 34.8 (8.94) | 40.0 (5.33) | 0.417 |
| Creatinine (mg/dL) | 0.785 (0.122) | 0.774 (0.141) | 0.783 (0.172) | 0.844 |
| Albumin (g/dL) | 4.65 (0.223) | 4.56 (0.180) | 4.62 (0.248) | 0.423 |
| Aspartate aminotransferase (UI/l) | 27.7 (12.3) | 23.5 (6.41) | 25.7 (8.26) | 0.733 |
| Alanine aminotransferase (UI/l) | 26.5 (17.1) | 23.1 (7.88) | 25.0 (10.9) | 0.992 |
| Cholesterol (mg/dL) | 197 (38.5) | 194 (38.1) | 200 (16.5) | 0.945 |
| Tryglicerides (mg/dL) | 94.2 (30.2) | 97.4 (33.1) | 112 (88.9) | 0.96 |
| HDL (mg/dL) | 63.4 (20.7) | 54.1 (8.94) | 60.8 (10.3) | 0.254 |
| LDL (mg/dL) | 115 (28.8) | 120 (31.3) | 117 (20.6) | 0.76 |
| APOA (mg/dL) | 159 (26.9) | 149 (13.5) | 159 (5.88) | 0.196 |
| APOB (mg/dL) | 91.5 (18.3) | 93.9 (18.3) | 95.0 (15.9) | 0.701 |
| IGG | 1070 (273) | 1040 (192) | 948 (163) | 0.539 |
| C-reactive protein (mg/dL) | 0.225 (0.302) | 0.171 (0.149) | 0.332 (0.610) | 0.673 |
| Prealbumin (g/dL) | 26.0 (4.46) | 24.0 (3.66) | 24.6 (6.65) | 0.45 |
| IgA (mg/dL) | 224 (101) | 231 (80.4) | 236 (119) | 0.813 |
| IgM (mg/dL) | 103 (48.8) | 95.0 (36.9) | 142 (52.7) | 0.095 |
| Cortisol (ug/dL) | 14.4 (3.43) | 16.4 (4.81) | 15.2 (7.03) | 0.469 |
| Insulin (uUl/mL | 8.93 (7.07) | 13.5 (23.5) | 5.13 (3.19) | 0.122 |
| HOMA | 2.10 (2.01) | 3.23 (6.46) | 1.15 (0.805) | 0.135 |
| Adrenaline (pg/ml) | 21.5 (8.34) | 16.6 (8.13) | 22.5 (5.21) | 0.004 |
| Noradrenaline (pg/ml) | 423 (171) | 382 (163) | 374 (157) | 0.779 |
| Dopamine (pg/ml) | 14.5 (5.98) | 13.6 (3.65) | 13.3 (3.88) | 0.97 |
| Serotonine (ng/ml) | 198 (64.5) | 241 (79.2) | 182 (70.8) | 0.068 |
| IgE (UI/ml) | 64.2 (137) | 49.2 (58.2) | 162 (282) | 0.684 |
| Length (cm) | 166 (8.41) | 165 (9.26) | 163 (5.34) | 0.613 |
| Weight (kg) | 75.9 (20.2) | 72.4 (8.03) | 70.6 (15.0) | 0.954 |
| BMI (kg/m2) | 27.1 (5.47) | 26.6 (2.95) | 26.4 (4.80) | 0.958 |
| Fat mass (%) | 30.6 (8.43) | 32.3 (10.7) | 30.3 (10.1) | 0.678 |
| Muscle mass (%) | 30.0 (4.32) | 29.3 (5.26) | 30.4 (5.24) | 0.62 |
| Visceral fat | 11.1 (6.38) | 9.63 (2.93) | 10.3 (5.85) | 0.974 |
| Waist (cm) | 91.8 (16.9) | 89.6 (8.48) | 86.7 (18.0) | 0.759 |
| Hip (cm) | 103 (9.70) | 102 (6.26) | 104 (8.12) | 0.874 |
| SBP (mmHg) | 131 (17.4) | 129 (19.9) | 128 (13.2) | 0.97 |
| DBP (mmHg) | 80.1 (13.1) | 77.9 (7.68) | 80.5 (12.5) | 0.581 |
| HR (pulse/min) | 67.1 (9.11) | 64.6 (9.11) | 65.5 (5.13) | 0.723 |

Table S1: Anthropometric and blood test values for each microbiota-based cluster, and p-values of the main effect of cluster. Continuous data are given as mean values (standard deviation) and categorical data as number and percentages. P-values by Kruskal-Wallis test for continuous variables, Chi^2^ test for binary/categorical variables.

Table S2

| Bacteria (genus) | Microbiota-based cluster main effect | Significant pairwise post hoc differences  (p-value) | | |
| --- | --- | --- | --- | --- |
|  |  | A-B | A-C | B-C |
| *Bacteroides* | F(2,48)=60.038, p=.000, η^2^_p_=.7.144, 1-β=1.000 | -.21200 (.000) |  | .28230 (.000) |
| *Prevotella 9* | F(2,48)=118.017, p=.000, η^2^_p_=.8.310, 1-β=1.000 |  | -.38280 (.000) | -.41690 (.000) |
| *Faecalibacterium* | F(2,48)=3.922, p=.026, η^2^_p_=.1.405, 1-β=.679 |  |  |  |
| *Mycobacterium* | F(2,48)=4.515, p=.016, η^2^_p_=.1.583, 1-β=.744 |  | -.00022 (.017) | -.00022 (.020) |
| *Holdemanella* | F(2,48)=4.273, p=.020, η^2^_p_=.1.511, 1-β=.719 |  | -.01685 (.024) | -.01887 (.011) |
| *Barnesiella* | F(2,48)=4.222, p=.020, η^2^_p_=.1.496, 1-β=.713 | -.00936 (.030) |  |  |
| *Ruminococcus (gnavus group)* | F(2,48)=3.832, p=.029, η^2^_p_=.1.377, 1-β=.668 | -.00056 (.032) |  |  |
| *Monoglobus* | F(2,48)=3.715, p=.032, η^2^_p_=.1.340, 1-β=.654 |  |  |  |
| *Unidentified (Ruminococcaceae)* | F(2,48)=3.210, p=.049, η^2^_p_=.1.180, 1-β=.586 |  |  |  |
| *Fournierella* | F(2,48)=3.814, p=.029, η^2^_p_=.1.371, 1-β=.666 |  |  |  |
| *Coriobacteriaceae UCG-003* | F(2,48)=4.022, p=.024, η^2^_p_=.1.435, 1-β=.691 |  | -.00044 (.018) | -.00045 (.020) |
| *Marvinbryantia* | F(2,48)=5.110, p=.010, η^2^_p_=.1.755, 1-β=.798 | .00041 (.013) |  |  |
| *Slackia* | F(2,48)=3.921, p=.026, η^2^_p_=.1.404, 1-β=.679 |  |  |  |
| *Libanicoccus* | F(2,48)=5.646, p=.006, η^2^_p_=.1.904, 1-β=.839 |  | -.00023 (.008) | -.00025 (.003) |
| *Lactiplantibacillus* | F(2,48)=3.800, p=.029, η^2^_p_=.1.367, 1-β=.664 |  | -.00019 (.020) | -.00017 (.044) |
| *Leuconostoc* | F(2,48)=4.059, p=.024, η^2^_p_=.1.447, 1-β=.695 |  | -.00001 (.017) | -.00001 (.020) |
| *Stenotrophomonas* | F(2,48)=4.515, p=.016, η^2^_p_=.1.583, 1-β=.744 |  | -.00005 (.017) | -.00005 (.020) |
| Others | F(2,48)=9.184, p=.000, η^2^_p_=.2.768, 1-β=.969 | .09777 (.000) |  |  |
| η^2^_p_: Effect size (partial eta square)  1-β: Observed power | |  |  |  |

Table S2: Microbiota genera that presented a significant main effect of cluster and the statistically significant post-hoc differences in relative proportion between the three microbiota-based clusters.

Table S3

| Frequency band | Brain ROI | Center MNI Coordinates  (X, Y, Z) | Microbiota-based cluster main effect | Significant pairwise post hoc differences (p-value) | | |
| --- | --- | --- | --- | --- | --- | --- |
|  |  |  |  | A-B | A-C | B-C |
| Theta  (4Hz-7Hz) | L A37mv | -31, -64, -14 | χ^2^(2)=11.231, p=.004 | - | -.427 (.015) | -572 (.002) |
|  | L A37lv | -38, -52, -14 | χ^2^ (2)=9.215, p=.010 | - | -223 (.028) | -.343 (.008) |
|  | L A5l | -33, -47, 50 | χ^2^ (2)=9.204, p=.010 | - | -1.149 (.007) | -.938 (.049) |
|  | L A5m | -8, -47, 57 | χ^2^ (2)=9.897, p=.007 | - | -1.482 (.005) | -1.208 (.032) |
|  | L A31 | -6, -55, 34 | χ^2^ (2)=10.871, p=.004 | - | -.679 (.004) | -.675 (.010) |
|  | L A23d | -4, -39, 31 | χ^2^ (2)=12.543, p=.002 | - | -.517 (.002) | -.524 (.004) |
|  | L A23v | -8, -47, 10 | χ^2^ (2)=10.714, p=.005 | - | -.298 (.004) | -.319 (.011) |
|  | L rLinG | -17, -60, -6 | χ^2^ (2)=10.582, p=.005 | - | -.327 (.012) | -.402 (.004) |
|  | R A31 | 6, -54, 35 | χ^2^ (2)=9.513, p=.009 | - | -.604 (.009) | -.629 (.013) |
|  | R A23d | 4, -37, 32 | χ^2^ (2)=10.125, p=.006 | - | -.462 (.005) | -.480 (.013) |
|  | R A23v | 9, -44, 11 | χ^2^ (2)=10.306, p=.006 | - | -.288 (.005) | -.308 (.012) |

Table S3: Brainnetome cortical areas that presented a significant main effect of microbiota-based cluster and the statistically significant post-hoc differences in spontaneous brain activity (source density) between the three microbiota-based clusters, for different frequency bands in the eyes closed condition. L: Left; R: Right.

Table S4

| Frequency band | Brain ROI | Center MNI Coordinates  (X, Y, Z) | Microbiota-based cluster main effect | Significant pairwise post hoc differences (p-value) | | |
| --- | --- | --- | --- | --- | --- | --- |
|  |  |  |  | A-B | A-C | B-C |
| Theta  (4Hz-7Hz) | L A31 | -6, -55,34 | χ^2^ (2)=9.681, p=.008 | - | -.447 (.030) | -.532 (.006) |
|  | L A23d | -4, -39, 31 | χ^2^ (2)=10.109, p=.006 | - | -.455 (.007) | -.449 (.009) |
| Low Alpha (7Hz-10Hz) | R IFS | 48, 35, 13 | χ^2^ (2)=9.131, p=.010 | -.332 (.009) | - | - |
| Mid Beta (18Hz-21Hz) | R A4ul | 34, -19, 59 | χ^2^ (2)=9.616, p=.008 | - | - | .567 (.012) |

Table S4: Brainnetome cortical areas that presented a significant main effect of microbiota-based cluster and the statistically significant post-hoc differences in spontaneous brain activity (source density) between the three microbiota-based clusters, for different frequency bands in the eyes open condition. L: Left; R: Right; IFS: Inferior frontal sulcus.
